# Is the burden of oral diseases higher in urban disadvantaged community compared to the national prevalence?

**DOI:** 10.1186/1471-2458-14-S3-S2

**Published:** 2014-11-24

**Authors:** Nasruddin Jaafar, Hina Hakim, Nor Azlida Mohd Nor, Asma Mohamed, Roslan Saub, Rashidah Esa, Jennifer Doss, Zamros Yuzadi Mohd Yusof, Norintan Ab-Murat, Noor Lide Abu Kassim, Hazreen Abdul Majid

**Affiliations:** 1 Community Oral Health Research Group, Faculty of Dentistry, University of Malaya, Kuala Lumpur, Malaysia; 2 Centre for Population Health (CePH), Department of Social and Preventive Medicine, Faculty of Medicine, University of Malaya, Kuala Lumpur, Malaysia

**Keywords:** Oral diseases, disadvantaged community, treatment needs.

## Abstract

**Background:**

The urban low income has often been assumed to have the greatest dental treatment needs compared to the general population. However, no studies have been carried out to verify these assumptions. This study was conducted to assess whether there was any difference between the treatment needs of an urban poor population as compared to the general population in order to design an intervention programme for this community.

**Methods:**

A random sampling of living quarters (households) in the selected areas was done. 586 adults over 19 years old living in these households were clinically examined using World Health Organization (WHO) Oral Health Survey criteria 4th edition (1997).

**Results:**

The overall prevalence of dental caries, periodontal disease, denture wearers and temporomandibular joint problems were 70.5%, 97.1%, 16.7% and 26%, respectively. The majority (80.5%) needed some form of dental treatment. The highest treatment needs were found in the oldest age group while the lowest were in the youngest group (19-29 years) (p = 0.000). The most prevalent periodontal problem was calculus; regardless of gender, ethnicity and age. Significantly more females (20.5%) wore prosthesis than males (11.1%) (p = 0.003). Prosthetic status and need significantly increased with age (p = 0.000). About one in four adults had Temporo-Mandibular Joint (TMJ) problems. Overall, it was surprising to note that the oral disease burden related to caries, prosthetic status and treatment need were lower in this population as compared to the national average (NOHSA, 2010). However, their periodontal disease status and treatment needs were higher compared to the national average indicating a poor oral hygiene standard.

**Conclusions:**

The evidence does not show that the overall oral disease burden and treatment needs in this urban disadvantaged adult population as higher than the national average, except for periodontal disease. The older age groups and elderly were identified as the most in need for oral health intervention and promotion. An integrated health intervention programme through a multisectoral common risk factor approach in collaboration with the Faculties of Medicine, Dentistry and other agencies is needed for the identified target group.

## Background

Non-communicable diseases (NCDs) are currently a global crisis. The burden of NCDs is high in low- and middle-income countries, contributed by poverty and also becoming a major barrier to development and achievement of the Millennium Development Goals (MDG) [1]. These income categories (low, middle and high), defined for World Health Organisation (WHO) member countries are based on the World Bank list of economies for the year 2011 (released in July 2012) [2]. According to the World Bank 2013, Malaysia is considered as a middle-income country [3]. One of the most neglected conditions in NCD is dental or oral diseases. The dental disease burden is a major public health problem in high income countries as well as a growing burden in many "low- and middle-income" countries [4]. The WHO oral health programme has worked hard to increase awareness in oral health care [5]. The policy of the WHO Global Oral Health Programme emphasized that oral health should be an integral and essential part of general health, and that oral health is an important determinant for quality of life [5]. Oral disease and its sequel causes pain and discomfort resulting in impairment of function and reduced quality of life.

More than half of the global population now lives in urban settings. Inequalities in health in urban settings to a great extent reflect inequities in economic, social and living conditions which have been the hallmark of most societies as a result of urbanization [6]. Thus, it is necessary to assess health and social needs of disadvantaged urban populations to improve their well being. It is estimated that more than 73% of the population in Malaysia now lives in urbanized areas according to the WHO data [7].

Malaysia is one of the countries that maintain a highly subsidized public health care system. However, oral disease data and treatment needs specifically targeted at the urban poor is scarce. The aim of this article was to report baseline data on oral health status and treatment needs of a disadvantaged urban adult population relative to the national prevalence, so that intervention programs in health promotion can be formulated. The objectives were: 1) to assess the prevalence of major oral diseases namely caries, periodontal disease, prosthetic status and TMJ disorders; 2) to assess the treatment needs of this population; and 3) to compare the prevalence in this population with the national oral health survey of adults data in 2010. With these data we hope to make the community aware about their problems, to empower them to change their lifestyle and control their environment and to propose specific interventional support for this disadvantaged group to the city authorities.

The following report is limited to analysis of oral health status and treatment needs of the study population in order to compare with the prevalence of oral disease burden reported in the adult national oral health survey 2010 (NOHSA, 2010). Although not directly comparable in terms of representativeness by ethnicity, the comparison with an established national reference database may provide an indication of the relative seriousness of the oral health problems among the urban poor. This oral health survey was part of a larger effort by University of Malaya to create a database that includes healthcare services utilization and expenditure data, food practices and nutrition, cancer, needs of the elderly, substance abuse and addiction, sexual and HIV/AIDS risk behaviour, in order to design appropriate intervention programs to improve overall health and well-being in the target population. Based on the overall situational analysis a long term integrated intervention and health promotion program for the urban poor will be implemented in cooperation with multi-sectoral stakeholders and relevant agencies.

## Methods

The study area consists of Kg Kerinchi and Pantai Dalam areas of Kuala Lumpur which is part of the Kuala Lumpur City Hall urban squatter resettlement program. This is a very densely populated area with good basic infrastructure such as water, electricity, sewage and community facilities for example recreational spaces, playground, shops and community hall. The population was originally illegal squatters of government land which was then redeveloped into high rise flats and sold to the squatters at a subsidized price. All living quarters within these areas constituted the sampling frame. A simple random sampling of households (LQ's) was done with a 95% confidence and 5% error to obtain a representative population of adults who lived in such households. All adults aged 19 years and above who lived in the LQ were included in the sample. Clinical oral health data were collected according to WHO standard clinical criteria [8] which included dental caries, periodontal status, prosthetic need and TMJ conditions. Prior to dental screening, training and calibration of examiners was carried out to achieve a best percentage agreement of 80%. We acknowledge that 85% agreement may be the ideal, however the authors have tried their best to conduct repeated calibration exercises amongst examiners and the final 80% agreement was the best effort which was contributed by erratic measurement of periodontal disease. However for all other conditions i.e. dental caries, TMJ and denture needs, the agreement was between 90-100 %.

The findings from this study were then compared with the National Oral Health Survey of Adults 2010 [9]. NOHSA 2010 is a national survey of Malaysian adults aged 15 years and above. The sample in NOHSA study was obtained using a two-stage stratified random sampling.

Data collected was checked for completeness followed by data cleaning prior to analysis. Analysis was done using SPSS version 16. Descriptive statistics such as frequency distribution and cross tabulation was done to determine the prevalence and treatment needs of the major oral diseases. The mean values (CIs) were derived for caries experience and its components (DMFT). The chi-square tests were used to assess the relationship between the major oral diseases, overall treatment needs and demographic characteristics. The significance level was set at 5% (p < 0.05).

## Results

A total of 586 adults 19 years and above living in the selected household were included. Table 1 shows the overall dental treatment need by demographic characteristics. There were more females (60%) than males (40%). Malays made up the majority (81.1%) of the sample, followed by Indians (17%) and Chinese (1.2%). The study sample was reclassified into age groups representing young adults, middle aged and the elderly. The majority of the population (80.5%) required some form of dental treatment regardless of gender (p = 0.362). However, the highest treatment needs were among Chinese (85.7%). The youngest adult group (19-29 years) was found to have significantly lower overall dental treatment needs than the older age groups (p = 0.000).

**Table 1:** Overall dental treatment need by demographic characteristics (n = 586)

Variable	No treatment need n (%)	Some form of dental treatment need n (%)		p-value
Overall treatment need	114 (19.5)	472 (80.5)	0.362	
**Gender**				
Male	50 (21.3)	185 (78.7)		
Female	64 (18.2)	287 (81.8)		
**Ethnicity**			*0.025	
Malay	84 (17.5)	396 (82.5)		
Indian	29 (29.3)	70 (70.7)		
Chinese	1 (14.3)	6 (85.7)		
**Age**			*0.000	
19-29	37 (35.2)	68 (64.8)		
30-39	18 (15.0)	102 (85.0)		
40-49	19 (13.6)	121 (86.4)		
50-59	25 (20.2)	99 (79.8)		
60 and above	15 (15.5)	82 (84.5)		

*Chi square test p < 0.05

Table 2 shows the caries experience (DMFT index) and its components. The overall caries prevalence was 70.5%. The mean DMFT was 12.7 (95% CI = 11.89-13.44) with missing teeth (MT) component contributing to most of it, i.e. mean = 8.73 (95% CI = 7.96-9.50) followed by decayed teeth (DT) component, i.e. mean = 2.66 (CI 95% = 2.40-2.93). The filled teeth (FT) component was the lowest contributor with mean = 1.27 (CI 95% = 1.08-1.46).

**Table 2:** Caries prevalence and experience (DMFT) and its components

Variable	Mean	95% CI		%
		**Lower**	**Upper**	

**DT**	2.66	2.40	2.93	
**FT**	1.27	1.08	1.46	
**MT**	8.73	7.96	9.50	
**DMFT**	12.70	11.89	13.44	
**Overall prevalence**				70.5

The caries experience (DMFT) increased with age in both males and females. The highest caries experience were observed in the elderly (60 years and above) in both males (19.53) and females (20.61); as compared to the youngest age group (19-29 years) with a DMFT of 5.09 and 4.90, respectively (Table 3). The missing teeth (MT) component contributed to most of the DMFT index and the trend also increased with age. The maximum DMFT was noted in the elderly (aged 60 and above) in both males (16.57) and females (18.53). Females had a higher mean missing teeth (MT) component across all age groups except in the young adults (19-29 years) age groups (p < 0.05).

**Table 3:** Caries prevalence and components by gender and age-group

Age group (years)	Gender	Prevalence n (%)	DT	FT	MT	DMFT
19-29	Male	25 (61.0)	2.39	0.63	*2.07	5.09
	Female	38 (59.4)	2.16	0.89	1.60	4.90
30-39	Male	37 (75.5)	3.22	1.37	2.86	7.45
	Female	55 (77.5)	2.82	1.65	3.51	7.82
40-49	Male	41 (80.4)	2.78	1.86	6.22	10.86
	Female	66 (74.2)	2.58	2.02	8.39	12.17
50-59	Male	35 (71.4)	2.50	0.35	12.30	13.83
	Female	49 (65.3)	2.25	0.19	16.06	18.29
60 and above	Male	35 (77.8)	2.96	0.44	16.57	19.53
	Female	31 (59.6)	2.00	0.50	18.53	20.61

*Chi square test p < 0.05

Table 4 shows the periodontal status by demographic characteristics. The study community showed a very high overall prevalence of periodontal disease (97.1%). Males had significantly higher prevalence of periodontal disease (95.9%) than females (92.7%). Calculus was more prevalent in females (49.5%) than males (44%). By ethnicity, Indians (51.7%) and Chinese (50.0%) had higher prevalence of calculus than Malays (46.4%). Young adults showed the highest prevalence of calculus (64.8%) compared to other age groups. The prevalence of deep pockets was 22.9% and 19.6% for pockets 4-5 mm and 6 mm or more, respectively.

**Table 4:** Periodontal status by demographic characteristics

Variable N = 537	Prevalence CPI > 1 n (%)	Healthy TN = 0 n (%)	Bleeding TN = 1 n (%)	Calculus TN = 2 n (%)	Pocket 4-5 mm TN = 3 n (%)	Pocket 6 mm or more TN = 4 n (%)
**Gender**						
Male	*218 (95.9)	9 (4.1)	6 (2.8)	96 (44.0)	53 (24.3)	54 (24.8)
Female	319 (92.7)	23 (7.2)	17 (5.3)	158 (49.5)	70 (21.9)	51 (16.0)
**Ethnicity**						
Malay	*441 (94.4)	25 (5.7)	16 (3.6)	207 (46.4)	105 (23.5)	93 (20.9)
Indian	87 (93.0)	6 (6.9)	7 (8.0)	45 (51.7)	17 (19.5)	12 (13.8)
Chinese	4 (75.0)	1 (25.0)	0	2 (50.0)	1 (25.0)	0
**Age**						
19-29	*105 (92.4)	8 (7.6)	7 (6.7)	68 (64.8)	16 (15.2)	6 (5.7)
30-39	119 (91.9)	9 (7.6)	7 (5.9)	53 (44.5)	32 (26.4)	18 (15.1)
40-49	135 (97.1)	4 (3.0)	5 (3.7)	63 (46.7)	34 (25.2)	29 (21.5)
50-59	104 (95.1)	5 (4.8)	2 (1.9)	41 (39.4)	25 (24.0)	31 (29.8)
60 and above	74 (91.9)	5 (8.1)	2 (2.7)	29 (39.2)	16 (21.6)	21 (28.4)
Overall Prevalence	97.1	6.0	4.3	47.3	22.9	19.6

*Chi square test p < 0.05

Table 5 shows the prosthetic status and needs by demographic characteristics. Significantly more females had prosthesis compared to males (p = 0.003) and this was also reflected in the need for prosthesis (p = 0.015). The prevalence of denture wearers were highest among Chinese (71.4%) as compared to the Malay and Indians (p = 0.000). Similarly, the highest prosthetic need was observed among the Chinese (57.1%). However the differences in prosthetic needs was not statistically significant (p = 0.328). By age group, the present study found that the prosthetic status and need increased with increasing age (p = 0.000) of the sample.

**Table 5:** Prevalence of prosthetic status and needs by demographic characteristics

	Prosthetic status			Prosthetic needs		
**Variable** **N = 586**	**No prosthesis** **n (%)**	**With prosthesis** **n (%)**	**p-value**	**No prosthetic need** **n (%)**	**With prosthetic need** **n (%)**	**p-value**

**Gender**						
Male	209 (88.9)	26 (11.1)		158 (67.2)	77 (32.8)	*0.015
Female	279 (79.5)	72 (20.5)	*0.003	201 (57.3)	150(42.7)	
**Ethnicity**						
Malay	400 (83.3)	80 (16.7)		300 (62.5)	180(37.5)	0.328
Indian	86 (86.9)	13 (13.1)	*0.000	56 (56.6)	43 (43.4)	
Chinese	2 (28.6)	5 (71.4)		3 (42.9)	4 (57.1)	
**Age**						
19-29	103 (98.1)	2 (1.9)		94(89.5)	11 (10.5)	*0.000
30-39	113 (94.2)	7 (5.8)		89 (74.2)	31(25.8)	
40-49	115 (82.1)	25 (17.9)	*0.000	71 (50.7)	69 (49.3)	
50-59	90 (72.6)	34 (27.4)		62 (50.0)	62 (50.0)	
60 and above	67 (69.1)	30 (30.9)		43 (44.3)	54 (55.7)	

*Chi square test p < 0.05

TMJ status by demographic characteristics is shown in Table 6. About one quarter of adults in the community (26%) had TMJ problems with an almost equal numbers of males and females being affected. However, the majority had no TMJ problems. There were no significant difference in the distribution of TMJ problems by ethnicity (p = 0.811) and age groups (p = 0.349).

**Table 6:** TMJ status by demographic characteristics

Variables (N = 586)	TMJ problems		p-value
	**No problems n (%)**	**With TMJ problems n (%)**	

**Overall prevalence**	434 (74.0)	152 (26.0)	
**Gender**			0.915
Male	175 (74.5)	60 (25.5)	
Female	260 (74.1)	91 (25.9)	
**Ethnicity**			0.811
Malay	354 (73.8)	126 (26.2)	
Indian	76 (76.8)	23 (23.2)	
Chinese	5 (71.4)	2 (28.6)	
**Age**			0.349
19-29	85 (81.0)	20 (19.0)	
30-39	87 (72.5)	33 (27.5)	
40-49	99 (70.7)	41 (29.5)	
50-59	89 (71.8)	35 (28.2)	
60 and above	75 (77.3)	22 (22.7)	

Chi square test p < 0.05

## Discussion

The present study was carried out on a poor urban population which was part of the Kuala Lumpur City Hall urban squatter resettlement program. Generally the access to oral health care for this community is not a problem because their residential flat location is very near to existing government dental clinics and the University of Malaya Dental Center which provides services at highly subsidized rates. However, we note that it was a very difficult and challenging task to administer the questionnaire and conduct oral examinations leading to several limitations. One of the limitations was that about 40% of the identified sample refused to participate in the oral examination. The final sample of 586 adults represented about 60% of the eligible sample. It is acknowledged that low response rates can potentially cause population estimates bias, both in precision and accuracy. This is due to the fact that the respondents and non respondents may differ in terms of their sociodemographic and health profile [10]. Since this study did not collect information about the non-respondents, the estimate bias cannot be ascertained. Weighted data was not used because the purpose of this survey was to estimate the actual needs of the community. With these limitations in mind, the present study revealed that although the overall treatment need of the study population was high, it was generally lower than the national average as reported by the National Oral Health Survey of Adults 2010 shown in Table 7[9].

**Table 7:** Comparison between data from the National Oral Health Survey of Adults (2010) and the study population (2012)

Variable	NOHSA (2010) Prevalence (%)	STUDY POPULATION (2012) Prevalence (%)
Dental Caries	89.5	70.5
Periodontal disease	94.0	97.0
Periodontal need	94.0	97.0
Prosthetic (denture wearers)	46.3	16.7
Prosthetic need	45.9	38.7
Overall treatment need	98.3	83.8

In terms of overall dental treatment needs, the study population had actually lower needs (83.8%) as compared to national prevalence (98.3%) [9]. This might be due to the better access to oral healthcare services in the capital city of Kuala Lumpur. However in the elderly age group 60 years and above, the treatment needs were very high (84.5%). Data on the overall treatment needs according to age group was not reported in the Malaysian survey to allow comparison [9]. However, the periodontal treatment need and the number of missing teeth were very high in the older age groups compared to the younger age groups. Compared to an Asian population in Thailand, the treatment needs of the elderly were only about 75% in Bangkok [11].

For caries, although the prevalence in the studied population (70.5%) was lower than the national average (89.5%), but in terms of severity, the urban poor population had more teeth affected by caries (i.e. DMFT 12.7) as compared to the national average DMFT 11.8 [9]. The highest contributor to the DMFT index in both populations was missing teeth. This implied that the use of restorative and preventive services in the study population was very poor. The results were very similar to a Vietnamese study where the high number of missing teeth and low number of filled teeth indicated that extraction was the most common treatment sought. This may be due to the fact that extraction is considerably cheaper and less time consuming than other dental procedures [12].

For periodontal disease, the overall prevalence in the present population was very (97.1%) much higher than the national average reported in NOHSA 2010, indicating that the standards of awareness of oral hygiene among adults were very poor. In terms of periodontal treatment needs, calculus was the most frequent condition especially among young adults and Indians. Many studies reported that periodontal diseases increased with age [13]. In the present study, the percentage of bleeding and calculus was higher among young adults; while severe periodontal disease, including shallow and deep pockets, were higher among older adults and elderly. When the present study was compared to the national adult oral health data, not much difference was observed. Similar trends were reported in other South-East Asian countries such as in Thailand and Southern Vietnam [11,12]. In the present study, there was a statistically significant difference between periodontal disease and gender. The prevalence of periodontal disease was higher among males. This may indicate females tend to have better oral hygiene practices and sought dental care more often than their male counterparts [13].

The present study indicated that prosthetic needs increased with age. The elderly group had the greatest need. However, the overall prosthetic needs of the study population (38.7%) were lower than the national data (46.3%) and were much lower when compared to a study among Thai adults in Bangkok (84.5%) [11]. We observed a substantial gap between normative prosthetic needs and status which indicates that most prosthetic needs in the study population were not met.

The prevalence of TMJ problems was higher (26%) in the study population as compared to a study in Thailand which reported only 7% with TMJ problems. We could not compare to the national prevalence of TMJ problems due to lack of data in NOHSA.

Although the present study found a high need for oral health care in this study population, nevertheless it was still lower than the national average reported in 2010 [9]. Ironically, although this urban disadvantaged community has excellent access to well-equipped government dental clinics at highly subsidized costs, encouraging them to utilize the services remains a major challenge. Thus, continuous implementation of community-based oral health promotion and prevention is needed, particularly to improve periodontal health status and service utilization. A multisectoral health promotion and intervention program should be encouraged to meet the needs and demands of this target population.

## Conclusion

The evidence does not show that the overall oral disease burden and treatment needs in this urban disadvantaged adult population are higher than the national average, except for periodontal disease. The older age groups and elderly were identified as the most in need for oral health intervention and promotion.

## List of abbreviations used

DT: Decayed teeth; DMFT: Decayed, Missing, And Filled teeth; FT: Filled teeth; LQ's: Living Quarters; MT: Missing teeth; MDG: Millennium Development Goals; NCD: Non-Communicable Diseases; NOHSA: National Oral Health Survey of Adults; TMJ: Temporo Mandibular Joint; WHO: World Health Organization.

## Competing interests

The authors declare that they have no competing interests.

## Authors' contributions

NJ, HH, NAMN were the principal investigators and responsible for writing the manuscript; RS, RE, JD, AM, ZY, NAM, NAK, HAM were involved in the overall study design, implementation, data and statistical analysis, proof reading and approving the manuscript.

## Authors' information

Nasruddin Jaafar, Hina Hakim, Nor Azlida Mohd Nor, Asma Mohamed, Roslan Saub, Rashidah Esa, Jennifer Doss, Zamros Yuzadi Mohd Yusof, Norintan Ab-Murat, Noor Lide Abu Kassim are lecturers in the Faculty of Dentistry University of Malaya, Kuala Lumpur Malaysia. Hazreen A Majid is lecturer in the Social and Preventive Medicine in Faculty of Medicine, University of Malaya, Kuala Lumpur Malaysia.
